# Studies Into β-Glucan Recognition in Fish Suggests a Key Role for the C-Type Lectin Pathway

**DOI:** 10.3389/fimmu.2019.00280

**Published:** 2019-02-26

**Authors:** Jules Petit, Erin C. Bailey, Robert T. Wheeler, Carlos A. F. de Oliveira, Maria Forlenza, Geert F. Wiegertjes

**Affiliations:** ^1^Cell Biology and Immunology Group, Wageningen University & Research, Wageningen, Netherlands; ^2^Department of Molecular & Biomedical Sciences, University of Maine, Orono, ME, United States; ^3^Graduate School of Biomedical Sciences and Engineering, University of Maine, Orono, ME, United States; ^4^Department of Research and Development, Biorigin Company, Lençóis Paulista, Brazil; ^5^Aquaculture and Fisheries Group, Wageningen University & Research, Wageningen, Netherlands

**Keywords:** β-glucan, primary macrophage, transcriptome analysis, RNAseq analysis, CTLD, C-type lectin-like domain, teleost, *Cyprinidae*

## Abstract

Immune-modulatory effects of β-glucans are generally considered beneficial to fish health. Despite the frequent application of β-glucans in aquaculture practice, the exact receptors and downstream signalling remains to be described for fish. In mammals, Dectin-1 is a member of the C-type lectin receptor (CLR) family and the best-described receptor for β-glucans. In fish genomes, no clear homologue of Dectin-1 could be identified so far. Yet, in previous studies we could activate carp macrophages with curdlan, considered a Dectin-1-specific β-(1,3)-glucan ligand in mammals. It was therefore proposed that immune-modulatory effects of β-glucan in carp macrophages could be triggered by a member of the CLR family activating the classical CLR signalling pathway, different from Dectin-1. In the current study, we used primary macrophages of common carp to examine immune modulation by β-glucans using transcriptome analysis of RNA isolated 6 h after stimulation with two different β-glucan preparations. Pathway analysis of differentially expressed genes (DEGs) showed that both β-glucans regulate a comparable signalling pathway typical of CLR activation. Carp genome analysis identified 239 genes encoding for proteins with at least one C-type Lectin Domains (CTLD). Narrowing the search for candidate β-glucan receptors, based on the presence of a conserved glucan-binding motif, identified 13 genes encoding a WxH sugar-binding motif in their CTLD. These genes, however, were not expressed in macrophages. Instead, among the β-glucan-stimulated DEGs, a total of six CTLD-encoding genes were significantly regulated, all of which were down-regulated in carp macrophages. Several candidates had a protein architecture similar to Dectin-1, therefore potential conservation of synteny of the mammalian *Dectin-1* region was investigated by mining the zebrafish genome. Partial conservation of synteny with a region on the zebrafish chromosome 16 highlighted two genes as candidate β-glucan receptor. Altogether, the regulation of a gene expression profile typical of a signalling pathway associated with CLR activation and, the identification of several candidate β-glucan receptors, suggest that immune-modulatory effects of β-glucan in carp macrophages could be a result of signalling mediated by a member of the CLR family.

## Introduction

Immunomodulation by β-glucans has been widely studied in teleost fish. Regardless of the administration route or fish species, β-glucan administration often has an immune stimulatory effect and can result in increased resistance to both viral and bacterial infections [reviewed by (1–3)]. For both, mammalian vertebrates (4, 5) and invertebrates (6, 7), specific mechanisms responsible for β-glucan recognition and/or downstream signalling have been described. Yet for teleost fish, despite the frequent application of β-glucans in aquaculture practice, the exact mechanisms underlying the induced effects are ill described.

In mammals, several non-exclusive pathways play a role in the recognition and down-stream signalling after β-glucan stimulation, however the best-described β-glucan receptor is Dectin-1, also known as C-type Lectin domain Family 7 member A (CLEC7A). Dectin-1 is a *C-type lectin super family V, NK cell receptors* transmembrane receptor with a single carbohydrate recognition domain (CRD) and a cytoplasmic tail containing one ITAM motif (8–10). Dectin-1 is predominantly expressed on cells from both the monocyte/macrophage and neutrophil lineages, where it acts as the major β-glucan receptor (11). Ligation of β-(1,2)-glucan to Dectin-1 is dependent on two specific amino acid residues in the CRD; tryptophan (W) and histidine (H), separated by a third residue (WxH motif) (12). Additionally, a tyrosine (Y) residue separated from histidine in the CRD by four residues (WxHxxxxY motif) is crucial for shaping the β-glucan binding cleft (13). This β-glucan binding cleft is formed by spatial arrangement of tryptophan, histidine and tyrosine in a triangular fashion resulting in a shallow hydrophobic surface groove, capable of accommodating and binding β-glucan chains through hydrophobic interactions (12–14). Also true for invertebrates, the same three residues are present in the binding domain of β-(1, 3)-glucan recognition protein (GNBP3), although not as a conserved motif (15). Dectin-1 signalling is activated following clustering in synapse-like structures formed within minutes after activation by particulate β-glucans (16). Following the interaction with β-glucan, Dectin-1 signals via Syk kinase and the adaptor protein Card9 to send a downstream signal through Bcl10 and Malt1 to the transcription factor NF-κB (17). Activation of NF-κB leads to an inflammatory profile typical of stimulation with β-glucans.

The presence or absence in fish genomes of Dectin-1, along with the entire superfamily V of CLRs (NK cell receptors), is debated (18). Although initially a cKLR from *Paralabidochromis chilotes* was described as a member of the superfamily V (19), and 28 distinct KLR loci identified a particular chromosomal region in Nile tilapia (*Oreochromis niloticus*) (20), a subsequent and thorough phylogenetic analysis suggested these receptors to be members of superfamily II rather than superfamily V (18). At the same time, it is important to realize that this phylogenetic analysis was primarily based on an early genome assembly of a single fish species, *Fugu rubripes* (18), indicating that the presence of true superfamily V CLR members in fish genomes has not been systematically investigated in other teleosts.

Functional and therefore indirect evidence of the presence of β-glucan receptor(s) in fish exists for at least Atlantic salmon (*Salmo salar*) macrophages, channel catfish (*Ictalurus punctatus*) neutrophils and seabream (*Sparus aurata*) leukocytes, based on observations that pre-treatment of these cell types with β-glucans reduces the uptake of yeast (*Saccharomyces cerevisiae*) glucan particles, zymosan or whole yeast cells (21–23). In carp, injection of β-glucans induced a complement receptor 3 (CR3)-dependent rosette formation of leukocytes and deposition of iC3b and C3d fragments on zymosan (24, 25). β-glucan was also shown to regulate the expression of several pattern recognition receptors including *tlr2* in primary macrophages of European eel (*Anguilla anguilla*) (26), regulation of *tlr3* and *cxc* receptors in common carp (27, 28), the purinergic receptor *p2x4* in Japanese flounder (*Paralichthys olivaceus*) (29) and *nod2* and *tlr2* in zebrafish (*Danio rerio*) (30). Although these genes were regulated by β-glucan stimulation, their involvement in the recognition of β-glucans remains to be confirmed. Oral intubation of Atlantic salmon (*S. salar*) with β-glucan resulted in the up-regulation of *syk* kinase and three salmon CLRs with one or two ITAMs and a single WxH motif (31, 32), alike the Dectin-1 architecture described above. We reasoned that macrophages could provide an informative starting point for investigating signalling pathways induced upon β-glucan stimulation. Indeed, primary macrophages of common carp have been shown to respond to curdlan and to zymosan depleted of Toll Like Receptor (TLR) stimulating properties; both considered Dectin-1-specific ligands in mammals (33). We therefore hypothesized that immune-modulatory effects of β-glucan in carp macrophages could be triggered by an unknown member of the CLR family, different from Dectin-1.

Here, we used primary macrophages of common carp for a whole transcriptome analysis of differentially expressed genes (DEG) induced by two different β-glucans. Analyses of gene ontology revealed comparable profiles and a clear regulation of the CLR pathway by both β-glucans. Subsequent investigation of the common carp genome for candidate β-glucan receptors identified a number of genes based on their architecture or expression profile, all encoding proteins with at least one C-type Lectin Domain (CTLD). Preliminary phylogenetic analysis of the CTLD sequences of candidate proteins showed no clustering with CTLD sequences of known group V members. Synteny analysis of the genome of zebrafish, a close relative of common carp, identified two CTLD-encoding genes with apparent conservation with mammalian CLR group V members, namely *clec4c* and *sclra*. Overall, our study identifies several teleost CLRs of interest for future functional studies aimed at further specifying the modulatory effects of β-glucans on the fish immune system.

## Methods

### Animals

European common carp (*Cyprinus carpio carpio* L.) of the R3 × R8 strain were used, which originate from a cross between the Hungarian R8 strain and the Polish R3 strain (34). Carp were bred and raised in the aquatic research facility of Wageningen University, Carus, at 20–23°C in recirculating UV-treated water and fed pelleted dry feed (Skretting, Nutreco) twice daily. All experiments were performed with the approval of the animal experiment committee of Wageningen University.

### *In vitro* Culture of Head Kidney-Derived Macrophages

Fish were anaesthetized with 0.3 g/l Tricaine Methane Sulfonate (TMS) (Crescent Research Chemicals, Phoenix, USA) in aquarium water buffered with 0.6 g/l sodium bicarbonate and bled via the caudal vein. Carp head kidney-derived macrophages were cultured for 6 days, as described previously (35), and will be referred to as macrophages.

### Macrophage Stimulation

Macrophages were harvested by placing culture flasks on ice for 15 min and by gentle scraping. Cell suspensions were centrifuged at 450 × *g* for 10 min at 4°C. Macrophages were resuspended in complete NMGFL (incomplete-NMGFL-15 medium supplemented with 2.5% heat-inactivated pooled carp serum and 5% bovine calf serum (Invitrogen Life Technologies) with 100 U/mL of penicillin and 50 μg/mL streptomycin) (35). Subsequently, macrophages were seeded in 24-well flat-bottom culture plates (Corning™ 3526, FischerScientific) at 1.5 × 10^6^ macrophages/300 μL per well. For stimulation of the cells, curdlan (C7821, Sigma Aldrich) (a high molecular weight linear polymer consisting of β-1-3-linked glucose residues from *Alcaligenes faecalis*) and MacroGard® [a cell wall preparation of *S. cerevisiae* comprising 91% β-glucan (Zilor, São Paulo, Brazil)] were used (36). β-glucans were prepared as previously reported (33). Cells were stimulated with β-glucan preparations at 25 μg/mL, a concentration at which both β-glucan preparations were previously shown to induce considerable nitric oxide production in carp macrophages (33). For each stimulus at least three independent cultures were used and each stimulation was performed in technical triplicate.

After 6 h of stimulation, three replicate wells were pooled and 4.5 × 10^6^ macrophages were lysed in 350 μL RLT buffer (QIAgen, Netherlands) and stored at −80°C until RNA extraction. Total RNA was extracted using the RNeasy Mini kit according to the manufacturer's protocol (QIAgen) including on-column DNase treatment with the RNase-free DNase set (QIAgen). RNA was stored at −80°C until use.

### Illumina Sequencing and Data Analysis

RNA quality and concentration was checked on a Bioanalyzer (Agilent 2100 total RNA Nano series II chip, Agilent). RNAseq libraries were prepared from 0.5 μg total RNA using the TruSeq® Stranded mRNA Library Prep kit according to the manufacturer's instructions (Illumina Inc. San Diego, CA, USA). Similar to the previous carp study (37), all RNAseq libraries were sequenced on an Illumina HiSeq2500 sequencer as 1 × 50 nucleotides single-end reads. Image analysis and base calling were performed using the Illumina pipeline. Using TopHat (version 2.0.5) (38), reads were aligned to the latest published genome assembly of common carp (BioProject: PRJNA73579) (37). For each independent sample at least 10 million raw reads were sequenced, on average 65% of the raw reads could be mapped to annotated genes of this carp genome assembly. Secondary alignments of reads were excluded by filtering the files using SAMtools (version 0.1.18) (39). Aligned fragments per predicted gene were counted from SAM alignment files using the Python package HTSeq (version 0.5.3p9) (40).

Differential gene expression was analysed using the bioinformatics package DESeq 2.0 (v1.22.2) (41) or edgeR (v3.24.3) (42, 43) from Bioconductor (v3.8) (44) in R statistical software (3.1.2) (45). Statistical analysis was performed using a paired design with unstimulated cells as control and performed for curdlan and MacroGard® independently (*n* = 3 independent cultures for curdlan and for MacroGard®). The paired design allowed for a better comparison between independent cultures, reducing noise generated by culture to culture differences (41). For DESeq 2.0, *p*-values were adjusted using Benjamini & Hochberg corrections for controlling false discovery rate and results were considered statistically significant when *p*-adjusted ≤ 0.05. For edgeR, genes were considered significantly regulated if both *p*-value ≤ 0.05 and FDR ≤ 0.05. Only genes identified as significantly regulated by both, DESeq 2.0 and edgeR, were used for subsequent analyses (Supplementary Tables 1, 2 for curdlan- and MacroGard® -DEGs, respectively). Venn diagrams were generated with the webtool from the University of Ghent Bioinformatics and Evolutionary Genomics group.^1^

### Gene Ontology Annotation and Enrichment Analysis

The common carp genome has been annotated against Ensembl zebrafish GRCz10 (37). Due to its tetraploid nature because of an additional genome duplication event (46–48), the common carp generally has two copies of each zebrafish gene. However, for gene ontology (GO) and KEGG analysis only single IDs were used, resulting in a dataset with unique Ensembl zebrafish IDs (curdlan DEGs *n* = 421 unique genes and MacroGard® DEGs *n* = 638 unique genes). Gene Ontology (GO) analysis was performed with GOrilla (49). Using differentially expressed genes as a target list and the entire list of annotated common carp genes as a background list, GO term enrichment was analysed (Supplementary Tables 3, 4 for curdlan and MacroGard®, respectively). FDR *q*-values were calculated by adjusting *p*-values using Benjamini & Hochberg method for controlling false discovery rate, GO terms were considered statistically enriched when FDR ≤ 0.05.

Independent KEGG analysis (50) was performed using the stable Ensembl zebrafish ID's of each differentially expressed gene in KOBAS v3.0 (51), using the well-annotated zebrafish genome as a reference list. KOBAS was run with Chi-square test and for FDR correction the Benjamini and Yekutieli method was used. Pathways were considered significantly over-represented if the corrected *p*-value was *p* ≤ 0.05. Recently, a zebrafish-specific KEGG pathway map for the CLR pathway was released (dre04625). As this map was not yet incorporated in the KOBAS analysis, we performed manual mapping using the “userdata mapping” feature on the zebrafish-specific KEGG pathway for C-type lectin receptor signalling.

### Genome Search for CTLD-Encoding Common Carp Sequences

The conceptual translation of all annotated carp gene sequences as submitted to NCBI (from here on referred to as carp proteins) [BioProject: PRJNA73579 (37)], was used to perform a Protein family (Pfam) domain search using CLC Main Workbench v8.0^2^ with the Pfam Database v31 (52). Proteins without a C-type lectin domain (CTLD) (PF00059) were filtered out. Subsequently, using the PatmatDB feature from EMBOSS in the public Galaxy server (v5.0.0) at Wageningen University and Research Centre (WUR, The Netherlands^3^, protein sequences containing an immunoreceptor tyrosine-based activation motif (ITAM) were identified using the signature YxxL/I sequence. Presence of a WxH motif within the CTLD was investigated using the PatmatDB feature from EMBOSS in Galaxy (v5.0.0) using the signature WxH. Alternatively, presence of a WxHxxxxY motif within the CTLD was investigated using the signature WxHx(1, 4)Y sequence, allowing for 1–4 random residues between histidine and tyrosine. Transmembrane regions of all proteins with at least one CTLD, were predicted using TMHMM Server v. 2.0^4^. These analyses highlighted a restricted number of proteins with one or more CTLDs and characterized the number of ITAM motifs, WxH motifs and transmembrane regions present in the conceptual translations. Subsets of candidate receptors were selected from the restricted number of proteins based on the following three criteria: (1) presence of a conserved WxH motif in the CTLD; (2) corresponding expression of ≥50 reads per kilobase million (RPKM) in unstimulated macrophages (*n* = 5); (3) differential regulation of expression in carp macrophages stimulated with β-glucans. Automatic annotation of candidate receptors was manually verified using BLASTx against the nr database from NCBI.

The CTLD sequences of identified candidate receptors were aligned with CTLD sequences from selected fish CLRs, selected mouse CLRs, several chicken CLRs (53) and with CTLDs from human CTLD-encoding genes (PF00059) present in Ensembl (GRCh38.p12) using MUSCLE v3.8 (54). In case of more than one CTLD sequence per protein, the first CTLD was designated 1, the next 2, and so on. Subsequently, Model Selection feature of MEGA-X (55) was used to calculate the most appropriate amino acid substitution model using all sites of the alignment as input data. The evolutionary history was inferred by using the Maximum Likelihood (ML) method based on the Whelan and Goldman model (56) allowing for Gamma distribution (+G) with four Gamma categories and using the rate variation model allowed for some sites to be evolutionarily invariable [(+I), 0.19% sites]. The bootstrap consensus tree inferred from *n* = 500 replicates was taken to represent the evolutionary history of the taxa analysed (57). Initial tree(s) for the heuristic search were obtained automatically by applying Neighbor-Join and BioNJ algorithms to a matrix of pairwise distances estimated using a JTT model, and then selecting the topology with superior log likelihood value. A discrete Gamma distribution was used to model evolutionary rate differences among sites (+G, parameter = 1.6778). The final alignment involved a total of *n* = 223 CTLD sequences.

As a fourth criterion for the identification of candidate receptors, conservation of synteny with the mammalian NK cell receptor cluster was added. Synteny analysis relied on genomic location data from NCBI and Ensembl Gene Summary databases. The GRCh38.p12, GRCm38.p6, and the Zv10 primary genome assemblies were used for human, mouse, and zebrafish genomic location data, respectively. For genomic location of carp genes the latest common carp assembly (37) was used.

## Results

### β-Glucan Stimulation of Macrophages Leads to Regulation of the CLR Signalling Pathway

**Figure 1:**
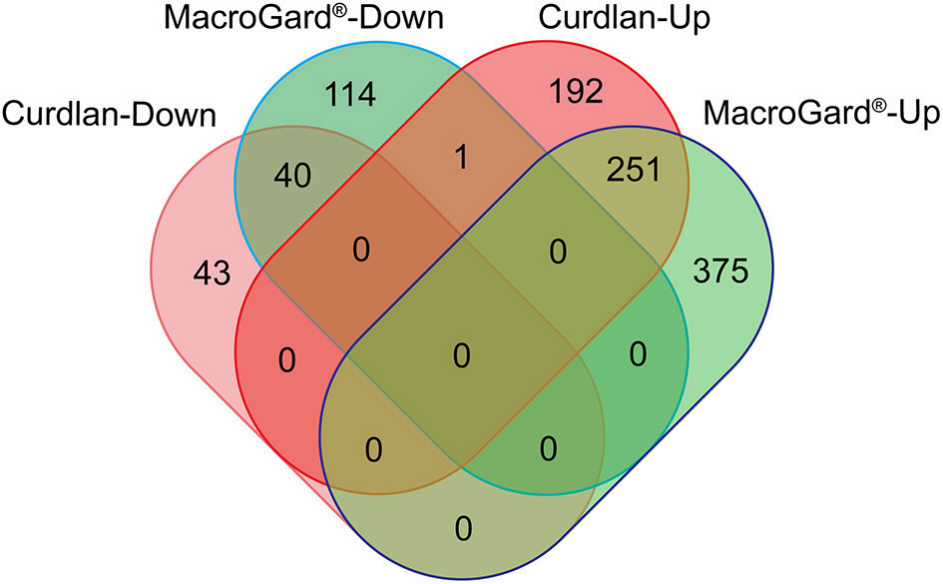
Venn diagram comparing differentially expressed genes (DEGs) regulated upon stimulation of common carp macrophages with β-glucans. Venn diagram of the DEGs regulated by curdlan (red) or MacroGard®(green). Macrophages were stimulated for 6 h with 25 μg/mL of either curdlan or MacroGard® and collected for RNAseq analysis.

Transcriptome analysis of macrophages stimulated with curdlan retrieved a total of *n* = 528 differentially expressed genes (DEGs) (Supplementary Table 1) of which almost 85% were up-regulated. Transcriptome analysis of macrophages stimulated with MacroGard® retrieved a total of *n* = 781 DEGs (Supplementary Table 2), of which almost 80% were up-regulated. Subsequent comparison of the expression profile of both DEG datasets revealed a comparable profile, with *n* = 291 DEGs that followed concordant expression patterns, only in one case discordant regulation was observed as up-regulation by curdlan and down-regulated by MacroGard® (Figure 1).

Automated gene ontology (GO) analysis of the two DEG datasets using GOrilla could map ~65% of the DEGs to GO terms. GO term enrichment analysis revealed 9 and 37 GO terms significantly enriched (FDR *q*-value ≤ 0.05) among curdlan-DEGs and MacroGard®-DEGs, respectively. For both datasets, the GO term with smallest FDR *q*-value was “Immune system process (GO:0002376).” Within the domain Biological Process, all significantly enriched GO terms in the curdlan-DEG dataset (*n* = 7), were also significantly enriched in the MacroGard®-DEG dataset (*n* = 25) (Supplementary Tables 3, 4), suggesting that the gene expression profile regulated by curdlan is also regulated by MacroGard®.

Automated annotation of the two DEG datasets to zebrafish KEGG pathways resulted in the mapping of the curdlan-DEGs to *n* = 92 different pathways and of the MacroGard®-DEGs to *n* = 112 different pathways. Four pathways were significantly over-represented in both DEG datasets, and a further nine unique pathways were significantly over-represented only in the MacroGard®-DEGs (Table 1). The overlap in the over-represented KEGG pathways in the two datasets supports the notion that in carp macrophages, the profile induced by curdlan is also induced by MacroGard®.

**Table 1:** Over-represented KEGG pathways following automated KEGG analysis of differentially expressed genes (DEGs) in macrophages stimulated with curdlan or MacroGard®.

**Pathway**	**Curdlan**	**MacroGard®**
Phagosome		X
Cytokine-cytokine receptor interaction	X	X
Lysosome		X
Apoptosis	X	X
Herpes simplex infection		X
Toll-like receptor signalling pathway		X
NOD-like receptor signalling pathway	X	X
VEGF signalling pathway		X
Arachidonic acid metabolism		X
Metabolic pathways		X
Phosphatidylinositol signalling system		X
ECM-receptor interaction	X	X
Adipocytokine signalling pathway		X

*Curdlan-DEGs (n = 356 genes) could be mapped to n = 92 pathways, of which n = 4 were significantly over-represented. MacroGard®-DEGS (n = 541 genes) could be mapped to n = 112 pathways of which n = 13 were significantly over-represented. KEGG analysis was performed with KOBAS v3.0 with Chi-square test and the Benjamini and Yekutieli method for FDR correction. Pathways were considered significantly over-represented at corrected p-value ≤ 0.05*.

**Figure 2:**
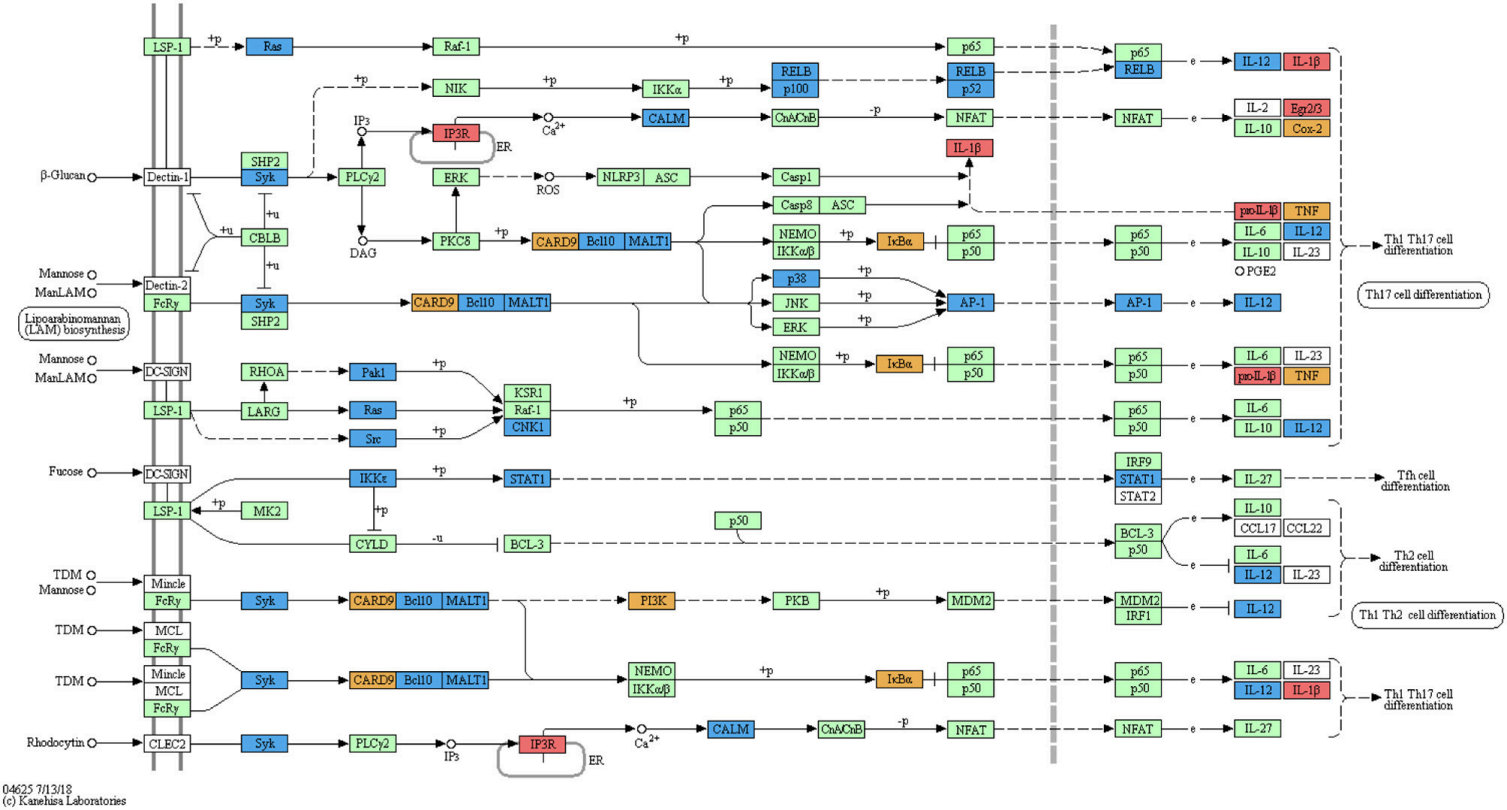
Regulation of the C-type lectin receptor signalling pathway by curdlan and MacroGard®. DEG datasets were manually mapped on the pathway-map dre04625^5^. The original reference pathway is indicated by white boxes, the organism-specific pathway is indicated by green boxes, confirming the presence of these genes in the zebrafish genome. Genes regulated after stimulation of carp macrophages with curdlan are indicated by red boxes, with MacroGard® by blue boxes, and by both curdlan and MacroGard® by orange boxes. The symbol O represents chemical compounds, DNA or other molecules. Black solid lines represent known molecular interaction or relation (▸: activating, |: inhibiting), dotted lines represent indirect links or unknown reactions, where +p, –p, +u, and –u represent phosphorylation, dephosphorylation, ubiquitination, and deubiquitination, respectively. Lines interrupted by e refer to gene expression interactions specifically.

Automated annotation did not include the zebrafish-specific KEGG map for the “C-type lectin receptor signalling pathway” (dre04625) which has become available only recently. Manual mapping of curdlan- and MacroGard®-DEGs showed regulation of a large number of genes associated with the C-type lectin receptor pathway (Figure 2). Further, not only end-products of the CLR signalling pathway, such as cytokines which are often shared among pathways, but also several upstream molecules, such as *card9, bcl10, malt1*, and *calm* were regulated (see Figure 2). The manual mapping suggests that in carp macrophages, both β-glucans induced expression of genes typically associated with C-type lectin receptor signalling.

### Search for Candidate Receptors

Following the above-described mapping of DEGs to the CLR pathway, we continued with screening all 50,527 conceptually translated carp proteins for the presence of at least one CTL domain (CTLD), narrowing down the search for candidate receptors to a total of *n* = 239 proteins. These CTLD-containing proteins were further characterized for the presence of ITAM sequences and transmembrane helices (data not shown). Studies have shown that the presence of a WxH motif or a WxHxxxxY motif in the carbohydrate binding region of Dectin-1 determines the β-glucan binding capacities of Dectin-1 (12–14). This criterion was used to further narrow down the search for candidate receptors, identifying a subset of *n* = 13 carp proteins with a WxH motif (Table 2). We could not identify proteins with a WxHxxxxY motif present specifically in their CTLD. Interestingly, in four WxH-containing proteins, we could also identify a transmembrane region and one or two ITAMs and thus a “complete” Dectin-1-like architecture. However, based on the RNAseq analysis, none of these candidates was significantly regulated by β-glucan stimulation or were constitutively expressed (higher than 10 reads per kilobase million) in unstimulated macrophages (Table 2), suggesting that these 13 candidates could not likely explain the functional responses to β-glucans in our current experimental set up.

**Table 2:** Candidate receptors with a WxH signature sequence in their CTL domain.

**Description**	**Accession number**	**CTLD**	**ITAM**	**TM**	**RPKM**	**cypCar**
**CD248 molecule, endosialin A precursor**	**NP_001092698.3**	**1**	**2**	**1**	**0.00**	**00001997**
C type lectin receptor A	NP_001117051.1	1	3 (4)	1	0.45 (0.00)	00016746, (00029396)
Aggrecan	BAJ61837.1	1	3	0	0.00	00023554
Novel protein with Lectin C-type domains precursor	NP_001093528.1	3	2	0	0.01	00031652
Novel protein similar to lectins	CAI21223.1	1 (2)	1	0	0.00	00037242, (00037243)
**Collectin 12**	**BAU33575.1**	**1**	**2**	**1**	**0.12**	**00040460**
C-type mannose receptor 2-like	XP_026052210.1	3 (1)	1 (4)	0	0.02 (0.00)	00041550, (00047187)
Secretory phospholipase A2 receptor-like	XP_026074237.1	2	1	0	0.00	00045110
**C-type lectin 1**	**AEH76769.1**	**1**	**1**	**1**	**6.67**	**00046286**
**Macrophage mannose receptor 1-like**	**XP_026074235.1**	**2**	**1**	**1**	**0.00**	**00048442**

*Description refers to the closest BLASTx hit upon blasting the identified genes of interest and accession numbers refer to protein sequences in NCBI. Number of conserved CTL domains (CTLDs), ITAM sequences and transmembrane domains (TM) in the protein sequence are included. RPKM refers to the average (n = 5) reads per kilobase million in unstimulated macrophages and is a measure for gene expression. cypCar codes identify common carp genes (Cyprinus carpio). Names or numbers in brackets refer to genes with identical BLASTx hits (likely owing to the additional genome duplication event in common carp). Proteins with architecture similar to Dectin-1 are highlighted in bold. Candidate receptors are ordered numerically by cypCar code*.

As the above-mentioned screening for WxH motif did not identify candidate receptors expressed or regulated in carp macrophages, we widened again our search and used constitutive expression or differential regulation as new criteria to narrow down the search for candidate receptors. Screening of all *n* = 239 CTLD-containing proteins for constitutive expression of their corresponding gene identified a subset of *n* = 12 genes that were expressed at an arbitrary threshold set at ≥ 50 Reads Per Kilobase Million (RPKM), corresponding to on average 1.5% of β-actin expression. Screening for regulation identified a subset of *n* = 6 candidate receptors as differentially expressed after stimulation with β-glucans, all of which overlapped with the expressed subset (Table 3). Of interest, all six were down-regulated by β-glucan stimulation and two out of the six candidate receptors (both Asialoglycoprotein receptor orthologues) were regulated by both β-glucans. The finding that these candidate receptors were all regulated could suggest involvement in the response to β-glucan simulation of carp macrophages.

**Table 3:** Candidate receptors expressed in carp macrophages.

**Description**	**Accession number**	**CTLD**	**ITAM**	**TM**	**RPKM**	**CRD**	**MG**	**cypCar**
**zgc:174904**	**NP_001170922.1**	**1**	**0**	**1**	**836**		**X**	**00031274**
**Asialoglycoprotein receptor 1-like isoform 1**	**XP_026096377.1**	**2**	**1**	**1**	**654**	**X**	**X**	**00032252**
Mannose receptor c type 1	ALS87701.1	2	2 (6)	0	467 (354)			00046879, (00034006)
**Asialoglycoprotein receptor 1-like isoform X2**	**XP_026098956.1**	**1**	**0**	**1**	**239**	**X**	**X**	**00032253**
Ladderlectin-like	XP_026129733.1	1	0	0	215			00046054
C-type lectin domain family 4 member E-like	XP_026087308.1	1	0	1	200			00044344
**C-type lectin domain family 4 member C**	**NP_001313501.1**	**1**	**1**	**1**	**165**		**X**	**00024225**
**Macrophage mannose receptor 1-like**	**XP_026142830.1**	**2 (5)**	**1 (7)**	**1**	**92 (60)**		**X**	**00047225, (00023855)**
CD209 antigen	XP_003197805.3	1	0	0	83			00049168
C-type lectin domain family 4 member E	XP_002660626.3	1	0	0	60			00014657

*Candidates were selected based on a minimal expression of ≥ 50 RPKM in unstimulated carp macrophages or differential expression following stimulation of macrophages with β-glucans. Description refers to the closest BLASTx hit, with associated NCBI protein accession code. Number of conserved CTL domains (CTLDs), ITAM sequences and transmembrane domains (TM) in the protein sequence are included. RPKM refers to the average (n = 5) reads per kilobase million in unstimulated macrophages and is a measure for gene expression. CRD and MG refer to differentially expressed genes in macrophages after stimulation with curdlan (CRD) or MacroGard® (MG) based on RNAseq analysis (highlighted in bold). cypCar codes identify common carp genes (Cyprinus carpio). Names or numbers in brackets refer to genes with identical BLASTx hits (duplicated genes). Candidate proteins are ordered based on their expression (RPKM)*.

**Figure 3:**
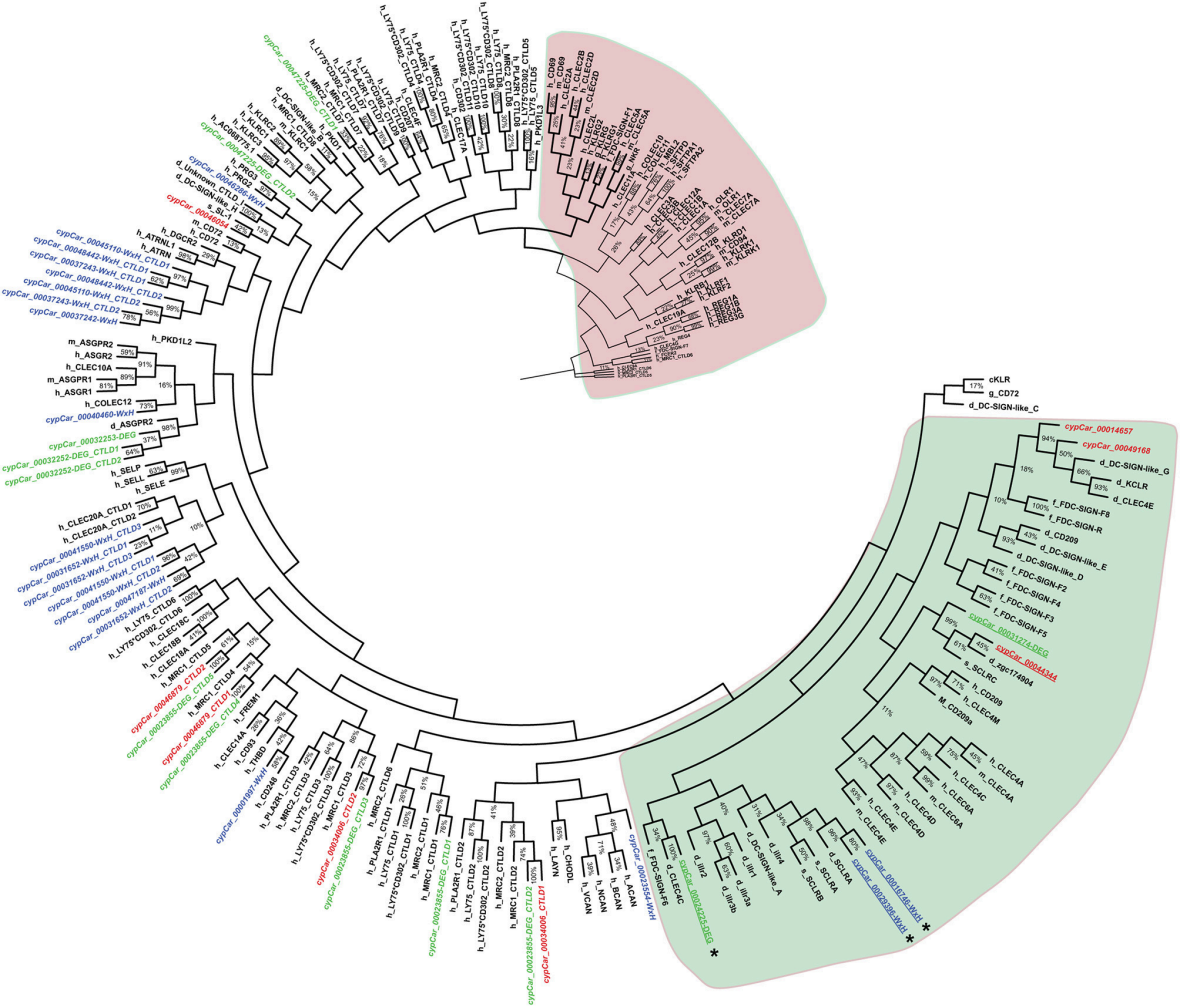
Molecular phylogenetic analysis of CTLD sequences from C-type lectin receptors by Maximum Likelihood. The large red or green-coloured clusters identify typical CLR superfamily group V members (red) and most group II members (green). Species are indicated as h_ for human, m_ for mouse, g_ for chicken, d_ for zebrafish, s_ for salmon f_ for fugu, and cypCar_ for carp. The percentage of replicate trees in which the associated taxa clustered together in the bootstrap test (500 replicates) are indicated, whereas values of partitions with bootstrap values lower than 10% are not shown. Carp CTLD sequences from candidate receptors (in italics) containing a conserved WxH motif are highlighted in blue, expressed in macrophages at RPKM ≥ 50 are highlighted in red and regulated by β-glucan stimulation of macrophages are highlighted in green. Asterisks (^*^) denote candidates identified independently with synteny analysis (see Figure 4). Underlined cypCar codes refer to candidates specifically addressed in the discussion section.

Combining the subsets of candidate β-glucan receptors identified based on criteria 1 (WxH motif), 2 (RPKM ≥ 50) and 3 (differential regulation) resulted in a total of *n* = 25 proteins of specific interest, containing *n* = 39 CTLD sequences. To investigate the phylogenetic relation between these candidates of interest and known CLR family members, CTLD sequences were aligned. The phylogenetic tree revealed a clear subdivision of the cypCars related to the carp CTLD proteins. None of the CTLD sequences from the identified candidates clustered with family V members, the group containing Dectin-1 (Figure 3). Instead, seven CTLD sequences from WxH motif-containing candidates (criterion 1) clustered with human CLEC20A, among human CLR group II members, and seven other WxH-candidates clustered together with human Attractin. Maybe not surprisingly, four of the constitutively expressed CTLD sequences (criterion 2) clustered with CTLD sequences from macrophage mannose receptors, group VI family. Six CTLD sequences from the differentially regulated subset (criterion 3) also clustered with macrophage mannose receptors, the other six CTLD sequences in this subset followed no specific clustering pattern. Overall, preliminary phylogenetic analysis suggests the absence of group V members in our set of candidate receptors.

### Synteny Analysis of Zebrafish and Carp CTLD-Encoding Genes

**Figure 4:**
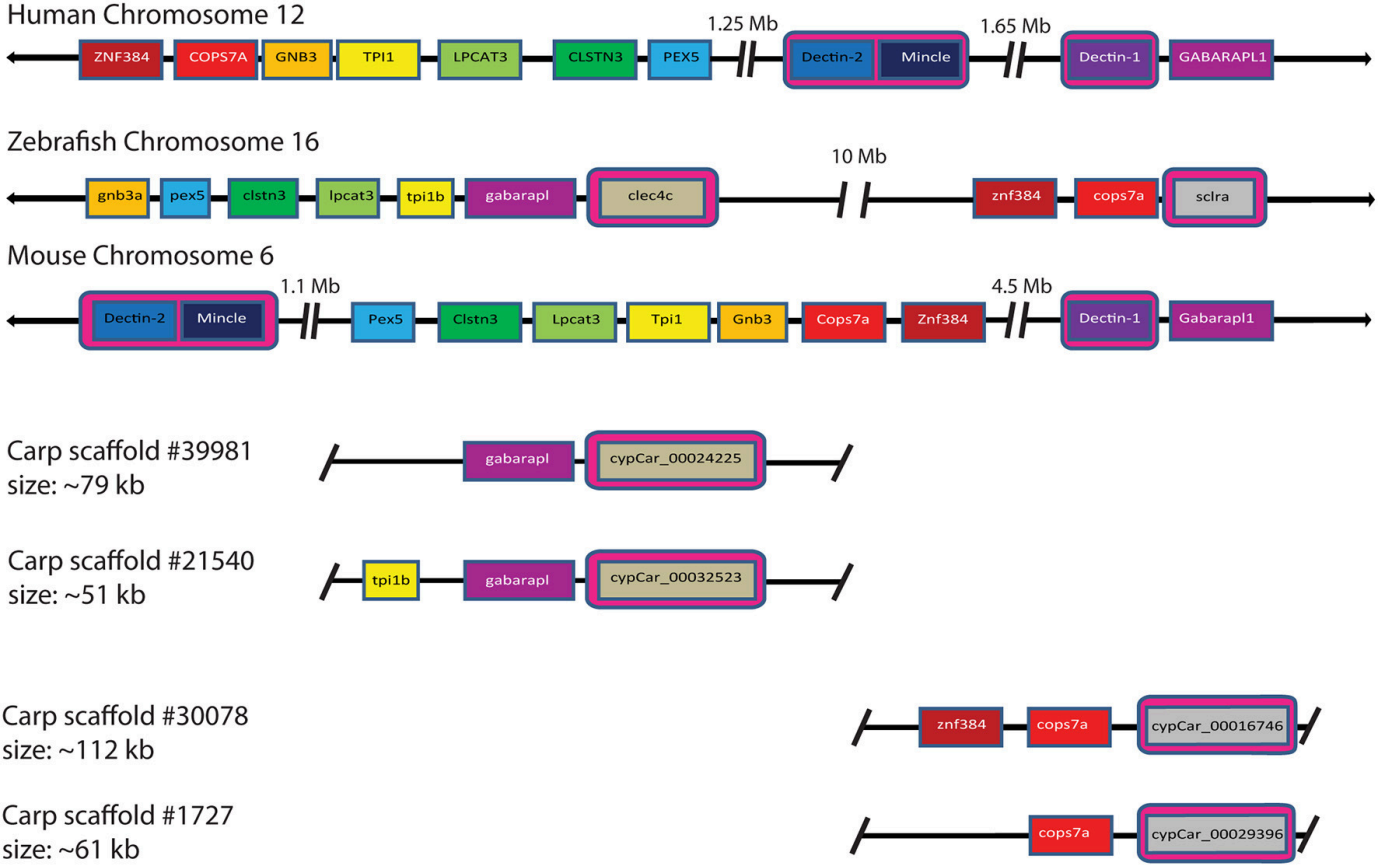
Synteny analysis of mammalian CLR group V cluster on human chromosome 12 and mouse chromosome 6 shows partial conservation with zebrafish chromosome 16 and four carp scaffolds. Partial conservation of synteny between zebrafish chromosome 16 and the mammalian CLR group V cluster can be observed. Among the syntenic genes, two zebrafish CLTD-encoding genes are located, *clec4c* (NCBI Gene ID: 563797) and *sclra* (NCBI Gene ID: 564061). Synteny of zebrafish *clec4c* and *sclra* with *Dectin-1, Dectin-2*, and *Mincle* is highlighted by pink boxes. Synteny of carp *cypCar*s is based on conservation of genes surrounding a CTLD-encoding gene for both *clec4c* and *scrla*. Large gaps between loci are indicated with breaks and chromosomal representations are not drawn to scale. Owing to the tetraploid nature of carp, two corresponding scaffolds for each zebrafish gene are shown.

Investigation of the carp CTLD containing proteins identified several candidates with architecture similar to mammalian Dectin-1. In mammalian genomes *Dectin-1* is located in the NK cell receptor cluster, which contains several CLRs. This NK cell receptor cluster shows conserved synteny between human chromosome 12 (hCHR12) and mouse chromosome 6 (mCHR6) (58). Conservation of synteny describes preservation of co-localization of genes on chromosomes of different species and might therefore serve as an additional criterion (criterion 4) for the identification for candidate receptors involved in the recognition of β-glucans. Synteny analysis can be best performed in a well-assembled genome. The genome assembly of zebrafish, a close relative of common carp, is among the best assembled genomes in teleost fish, including large chromosome scaffolds that can be used for synteny analysis, in contrast to the carp genome which is still largely fragmented into small scaffolds (37, 47). The NK cell receptor cluster in human includes, among others, *DECTIN-1, DECTIN-2*, and *MINCLE*. Regions surrounding the NK cell receptor cluster on hCHR12 and mCHR6 showed conserved synteny with regions on zebrafish chromosome 16 (zCHR16), based on co-localization of *pex5, clstn3, lpcat3, gnb3, cops7a*, and *znf384* (Figure 4). Intriguingly, this region of zCHR16 also includes two CTLD-encoding genes, *clec4c* (NCBI Gene ID: 563797), and *sclra* (NCBI Gene ID: 564061), highlighting them both as genes of interest for our study.

Further investigation of synteny between the zebrafish region of interest on zCHR16 and carp scaffolds revealed partial conservation of synteny with four scaffolds. Two of these scaffolds contained *cypCar_00024225* and *cypCar_00032523*, putative paralogues of zebrafish *clec4c*; of which *cypCar_00024225* is a CTLD-encoding gene, already identified in this study as candidate receptor based on its regulation in macrophages after stimulation with MacroGard® (criterion 3). The two other scaffolds contained *cypCar_00016746* and *cypCar_00029396*, putative paralogues of zebrafish *sclra*; both of which are CTLD-encoding carp genes, already identified in this study as candidate receptors based on their conserved WxH-motif (criterion 1). The synteny analysis provided a fourth criterion, additional to the previously formulated criteria (WxH motif, constitutive expression and differential regulation), to identify candidate receptors for β-glucan recognition. A Venn diagram, graphically representing the different subsets of candidate β-glucan receptors identified by the four criteria can be found Supplementary Figure 1.

## Discussion

Primary macrophages of common carp had previously been shown to respond to prototypical Dectin-1 ligands, which led to the hypothesis that the CLR pathway must play an important role in the recognition of β-glucans in carp macrophages. In our approach, we used head kidney-derived carp macrophages as a starting cell population to test our hypothesis and investigate activation of the CLR pathway upon stimulation with β-glucans. Indeed, pathway analysis of differentially expressed genes confirmed our hypothesis that β-glucans regulate a downstream signalling pathway typical of CLR activation. Further, we could identify in the transcriptome of β-glucan-stimulated carp macrophages, several differentially expressed genes with a C-type lectin domain. These data are of high interest for further functional studies on the mechanisms underlying β-glucan-induced immunomodulation in teleost fish.

We used two different β-glucans: curdlan, a linear polymer of β-(1,3)-linked glucose and considered a Dectin-1-specific ligand, and MacroGard®, a branched polymer of β-(1,3/1,6)-glucose widely-applied as feed additive in aquaculture. Overall, MacroGard® regulated a higher number of differentially expressed genes than curdlan, possibly owing to differences in purity, source, degree of polymerization, and nature of the glycosidic bonds in the β-glucans (59). Regardless of the extent of gene regulation, manual mapping of the DEGs revealed a clear regulation of the CLR signalling pathway (KEGG) for both β-glucans. Indeed, up-regulation of homologues of all three players of the *card9-Bcl10-Malt1* complex, previously shown to play a crucial role in β-glucan signalling through the CLR pathway (17), strongly supports regulation by the CLR pathway. We continued our study by identifying candidate genes encoding for proteins with one or more C-type Lectin Domains (CTLD), which could be of potential interest with respect to recognition of β-glucans, using a recently published database of RNAseq-validated gene predictions for carp (37). We used four criteria to identify candidate receptors in carp macrophages: (1) conservation of the glucan binding WxH-motif in the CTLD; (2) constitutive expression higher than 50 RPKM; (3) differential regulation upon stimulation with β-glucans; (4) conservation of synteny with mammalian NK cell receptor cluster.

Based on criterion 1 (conserved WxH motif) we identified two candidates (*cypCar_00016746* and *cypCar_00029396*) of which the CTLD clustered together with known CTLD sequences from zebrafish and from Atlantic salmon, known as *salmon C-type lectins sclra* and *sclrb* (31). Although the carp *sclr* paralogues were not constitutively expressed in macrophages (criterion 2), both salmon *sclr*s have been associated with the response to β-glucans (32). Interestingly, the mammalian WxHx[4]Y motif was not conserved, however a motif with five rather than four residues (WxHx[5]Y) separating histidine from tyrosine was conserved between all sequences. All three residues are considered crucial to form the β-glucan binding cleft of mammalian Dectin-1 (13), and also present in invertebrate β-glucan binding proteins (GNBP3), but not as a WxHx[5]Y motif (13, 15). Although not constitutively expressed in macrophages, carp *sclr* could well play a role in β-glucan binding in other cell types.

Based on criterion 2 (constitutive expression ≥ 50 RKPM), we identified a further 13 candidate receptors, of which six were differentially regulated (criterion 3). Without exception, all six genes were down-regulated upon stimulation with β-glucans, which could possibly be explained by a need to restrict *de novo* protein synthesis and duration of signalling to prevent over-stimulation (60–63). Possibly, analysis of protein and/or gene expression at different time points could show up-regulation. In Atlantic salmon, three *sclr*s were up-regulated 7 days after oral administration of MacroGard®, concomitantly with *syk* kinase (32). We similarly noticed a concomitant regulation of *syk*, suggesting the expression of CTLD-encoding genes and *syk* is co-regulated. No matter what, the observed modulation of gene expression strongly suggests involvement of CLR family members upon recognition of β-glucans by carp macrophages.

Based on criterion 3 (regulation of gene expression), we identified several additional candidate receptors. Among these, the CTLD-encoding gene with the highest expression and regulation in carp macrophages (*cypCar_00031274*), clustered together with three other genes of interest: (i) a CTLD-encoding gene already identified in this study based on criterion 2 (*cypCar_00044344)*, (ii) an unknown zebrafish full length cDNA (*zgc174904*) encoding a protein with a CTLD, a transmembrane helix and a WxHx[5]Y motif just outside the borders of the CTLD and (iii) an Atlantic salmon C-type lectin receptor-c (*sclrc*), different from the ones previously mentioned in criterion 1. The salmon *sclr* genes were first identified in (suppression subtractive) EST libraries (31), while a follow up study revealed up-regulation of all three *sclr* genes after oral intubation of Atlantic salmon with MacroGard® (32). The salmon *sclrc* gene contains a WxH motif within the CTLD while the carp candidate genes (both *cypCar_00031274* and *cypCar_00044344*) contain a WxHx[5]Y. Interestingly, the latter motif was found just outside the boundaries predicted for a CTLD, which suggests that further manual scrutiny of predicted domains in fish sequences could expand the list of currently identified domains of interest. Overall, the fact that closely-related *sclr* genes were identified as candidate receptors in both, salmon and carp, suggests a potential role of this CTL receptor in the response to β-glucans in fish and supports a need for its further characterization of function.

Based on criterion 4 (synteny in the zebrafish genome), we identified two CTLD-encoding genes of interest; putatively named *clec4c* and *sclra*. Three out of four corresponding duplicates at syntenic regions of the carp genome were already identified as genes of interest based on the selection criteria discussed above. This means that next to the *sclr* genes, we could identify *clec4c* as another gene of interest deserving further attention as candidate receptor for β-glucan. Taken together, our broad NGS approach helped us describe a clear regulation of the CLR pathway and identify a number of CTLD-containing candidate receptors for β-glucan binding. As proteins, these receptors would form a good starting point for future sugar binding assays and for further functional characterization with e.g., glycome microarrays, a high-throughput method devised to analyse β-glucan-binding proteins through an oligosaccharide microarray, followed by mass-spectrometric sequencing (64, 65). Altogether, the candidates discussed in this study should help pave the way to future functional studies that could ultimately lead to the identification of β-glucan receptor(s) in fish.

## Ethics Statement

This study was carried out in accordance with good animal practice as defined by the European Union guidelines for the handling of laboratory animals (http://ec.europa.eu/environment/chemicals/lab_animals/home_en.htm). Animal work in Wageningen University was approved by the local experimental animal committee (DEC number: 2017.W-0034).

## Author Contributions

JP, MF, and GW contributed to the design of the experiments, acquisition of samples, and analysis of data. GW acquired funding. RW and EB contributed with the phylogenetic and synteny analysis. CdO contributed with reagents, materials, and analysis tools. JP, RW, MF, and GW wrote the manuscript.

### Conflict of Interest Statement

CdO is an employee of Biorigin Company. The remaining authors declare that the research was conducted in the absence of any commercial or financial relationships that could be construed as a potential conflict of interest.

## Footnotes

^1^http://bioinformatics.psb.ugent.be/webtools/Venn/

^2^https://www.qiagenbioinformatics.com

^3^http://galaxy.wur.nl

^4^http://www.cbs.dtu.dk/services/TMHMM/

^5^https://www.genome.jp/kegg-bin/show_pathway?dre04625
