# Supplementary material for: Studies Into β-Glucan Recognition in Fish Suggests a Key Role for the C-Type Lectin Pathway
Source: Front Immunol. 2019 Feb 26;10:280. doi: 10.3389/fimmu.2019.00280 (PMC6400144; doi:10.3389/fimmu.2019.00280)
Supplement: Supplementary file 5 [file Image_1.pdf]

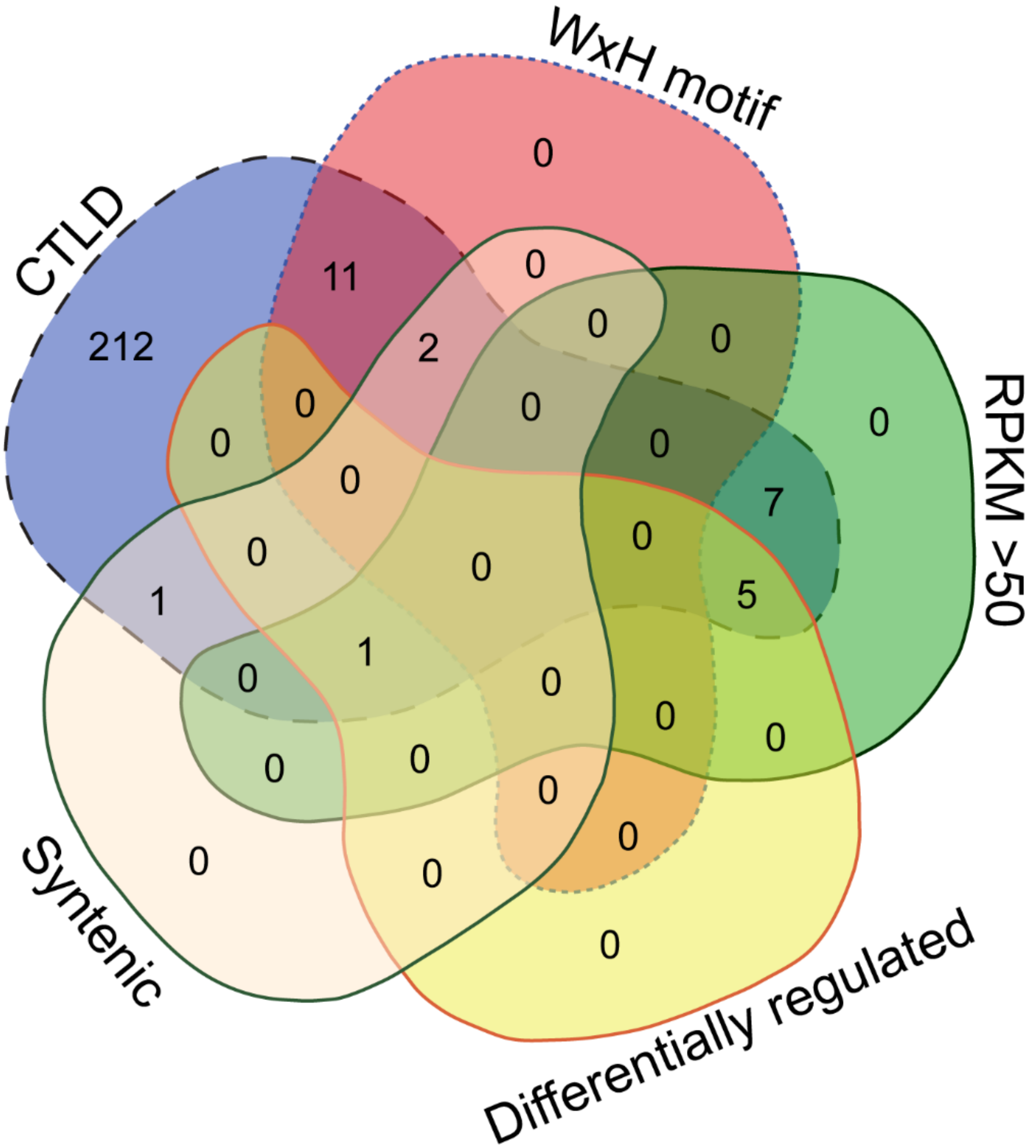

Supplementary figure 1: Venn diagram visualizing all C-type lectin domain (CTLD) encoding genes and the separate filtering criteria for potential β-glucan receptor candidates. All CTLD-encoding genes are identified with a protein family (Pfam) search of the protein sequence (blue shape). Four independent criteria were used to highlight candidate β-glucan receptors genes: 1) conservation of the glucan binding WxH-motif in the CTLD (red shape); 2) expression higher than 50 RPKM in carp macrophages (green shape); 3) differential regulation by stimulation of carp macrophages with β-glucans (yellow shape); 4) CTLD-encoding genes located in a region with conserved synteny to the mammalian CLR group V cluster are highlighted (orange shape).
